# Unveiling APOL1 haplotypes in a predominantly African-American cohort of kidney transplant patients: a novel classification using probe-independent quantitative real-time PCR

**DOI:** 10.3389/fmed.2024.1325128

**Published:** 2024-04-10

**Authors:** Murat Dogan, Christine Watkins, Holly Ingram, Nicholas Moore, Grace M. Rucker, Elizabeth G. Gower, James D. Eason, Anshul Bhalla, Manish Talwar, Nosratollah Nezakatgoo, Corey Eymard, Ryan Helmick, Jason Vanatta, Amandeep Bajwa, Canan Kuscu, Cem Kuscu

**Affiliations:** ^1^Transplant Research Institute, Memphis, TN, United States; ^2^Department of Surgery, College of Medicine, University of Tennessee Health Science Center, Memphis, TN, United States; ^3^Methodist Hospital, Memphis, TN, United States; ^4^HCA Florida Largo Hospital, Largo, FL, United States

**Keywords:** APOL1 gene, APOL1 G0/G1/G2 gene variants, kidney tranplantation, qPCR (quantitative PCR), kidney functions

## Abstract

**Introduction:**

Apolipoprotein-L1 (APOL1) is a primate-specific protein component of high-density lipoprotein (HDL). Two variants of APOL1 (G1 and G2), provide resistance to parasitic infections in African Americans but are also implicated in kidney-related diseases and transplant outcomes in recipients. This study aims to identify these risk variants using a novel probe-independent quantitative real-time PCR method in a high African American recipient cohort. Additionally, it aims to develop a new stratification approach based on a haplotype-centric model.

**Methods:**

Genomic DNA was extracted from recipient PBMCs using SDS lysis buffer and proteinase K. A quantitative PCR assay with modified forward primers and a common reverse primer enabled us to quantitatively identify single nucleotide polymorphisms (SNPs) and the 6-bp deletion. Additionally, we used Sanger sequencing to verify our QPCR findings.

**Results:**

Our novel probe-independent qPCR effectively distinguished homozygous wild-type, heterozygous SNPs/deletions, and homozygous SNPs/deletions, with at least 4-fold differences. A high prevalence of APOL1 variants was observed (18% two-risk alleles, 34% one-risk allele) in our recipient cohort. Intriguingly, no significant impact of recipient APOL1 variants on transplant outcomes was observed up to 12-month of follow-ups. Ongoing research will encompass more time points and a larger patient cohort, allowing for a comprehensive evaluation of G1/G2 variant subgroups categorized by new haplotype scores, enriching our understanding.

**Conclusion:**

Our cost-effective and rapid qPCR technique facilitates APOL1 genotyping within hours. Prospective and retrospective studies will enable comparisons with long-term allograft rejection, potentially predicting early/late-stage transplant outcomes based on haplotype evaluation in this diverse group of kidney transplant recipients.

## Introduction

Apolipoprotein L1 (APOL1) is a primate-specific apolipoprotein-L family member and is considered a minor component of high-density lipoprotein (HDL). It plays a role in lipid exchange and the transport of cholesterol from peripheral cells to the liver. The APOL1 gene is on the q-arm of human chromosome 22, comprises five operational domains, along with the other five APOL genes (1–5), and encodes several different transcript variants (6). APOL1 is primarily synthesized in the liver and found in several tissues such as the liver, heart, lung, podocytes and proximal tubules in the kidney (1, 2).

**Figure 1:**
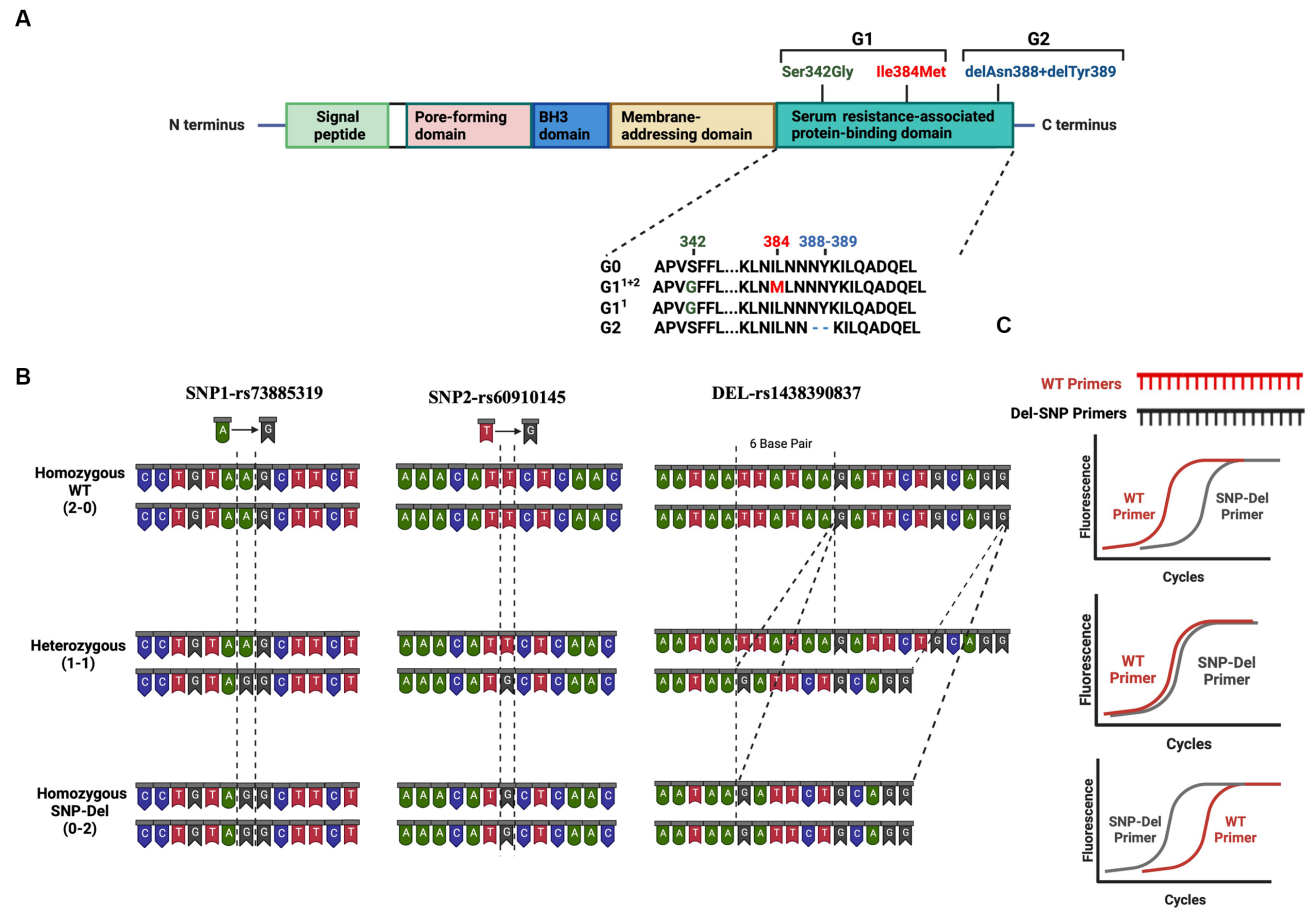
Risk Variants of the APOL1 gene **(A)** Graphical representation of the APOL1 protein showing its domains and the location of the G1 and G2 variants in the SRA domain **(B)** Representation of the base changes at individual SNP and deletion sites. DNA structures for homozygous wild-type (2–0), heterozygous (1–1), and homozygous SNP/deletion (0–2) variants are shown in three panels. **(C)** Predicted results of qPCR analysis using our probe-independent real-time quantitative PCR approach. This figure has been generated with Biorender program.

APOL1 is recognized as a secreted protein that travels through the bloodstream and assembles into a complex referred to as a trypanosome lytic factor (TLF), which is synthesized from the serum resistance-associated binding domain (SRA) (3) with high-density lipoprotein 3 (HDL3) and the hemoglobin-binding, haptoglobin-related protein (HPR). Within this complex, the APOL1 protein functions as the primary lytic element (3). TLF provides defense for humans, gorillas, baboons, and select individuals against prevalent African trypanosomes, a condition known as African sleeping sickness, which is caused by *Trypanosoma brucei* (4, 5, 7). Two genetic variants of APOL1 emerged within the human population of sub-Saharan Africa and rapidly disseminated across all African populations due to their ability to offer heightened defense against the virulent subspecies of trypanosomes known as *T. brucei rhodesiense* (8, 9). These gene variants are G1 coding variants which encompass two non-synonymous single nucleotide polymorphisms (SNPs), while G2 involves an in-frame deletion of the amino acids N388 and Y389 (Figure 1A).

Patients carrying one or two risk alleles are more likely to experience kidney dysfunction, potentially compromising transplant outcomes compared to patients without these risk alleles (10). These variants cause collapsing glomerulopathy, podocytopathy, and tubulopathy via mechanisms for podocyte injury including cationic pore-forming ability, altered autophagy, and direct toxicity (11–13). The toxicity of risk variants is associated with background haplotypes. Based on these 2 SNPs and 1 deletion, all risk alleles can be classified as no-risk allele (G0/G0), one-risk allele (G0/G1, G0/G2) and two-risk allele genotypes (G1/G1, G1/G2, G2/G2) (14). Nevertheless, it is important to consider that offspring inherit one allele from four potential haplotypes from each parent. The prior division of APOL1 genotypes, such as 2 groups for one-risk allele and 3 groups for two-risk alleles, could result in misinterpretation when assessing the impact of APOL1 variants and categorizing APOL1-related kidney diseases. Thus, a more nuanced subgrouping within each risk category may be necessary, based on an analysis of individual SNPs at the G1 position and their combinations with deletion variants at the G2 position (Figure 1A).

Genetic testing and probe-reliant PCR screening are currently used for the detection of variants, alongside costly and labor-intensive mass spectrometry methods. This underscores the need for quicker, more cost-effective ways to identify variants, especially for screening patients for potential risk in clinical settings. To address this, we have developed a probe-independent quantitative polymerase chain reaction (qPCR) technique for identifying APOL1 variants (Figure 1B), primarily aimed at our transplant cohorts, which mainly consist of African-American patients.

## Methods

### Study design and participants

This study included samples from patients who had kidney transplant surgery. Briefly all patients admitted to the Transplant Institute at Methodist University Hospital from May 2021 to May 2023 were considered for inclusion in this prospective study. The study comprised patients who had elective transplant surgery. A total of 171 patients were included in our sample biobank upon being given written informed consent for participation. Samples were collected before and after surgery for the patients and after receiving the organ from the donors. Following hospital discharge, patients were prospectively followed up for 12 months (weeks 1, 2, 3, 4, and months 2, 3, 6, 9 and 12). All biochemical and patient outcomes were recorded. Patients were treated according to the immunosuppression protocol of the Methodist Transplant Institute before and after transplantation. Immunosuppression induction was performed with 4.5-6 mg/kg of thymoglobulin and 500 mg of steroids before the operation. Patients were maintained under triple immunosuppression including tacrolimus, CellCept and prednisone. The tacrolimus target levels for the patients are 8-10 ng/mL for the first 3 months and 6–8 ng/mL for the 3 to 12 months after kidney transplantation.

The study protocol was approved by the local ethics committee of The Institutional Review Board of the University of Tennessee Health Science Center (IRB Approval Number: 20-07838-XP). All clinical examinations were conducted in accordance with the Declaration of Helsinki. Demographic characteristics and laboratory findings were collected.

### DNA isolation from patient blood samples

We isolated genomic DNA from PBMC samples from recipients. Blood samples were collected from patients before surgery in tubes with K2 EDTA additive to obtain PBMCs using the Ficoll gradient method. Blood samples were initially spun at 2200 G for 30 min and separated from plasma. After this step, they were resuspended with PBS and slowly added to an equal amount of Ficoll solution in a 15 mL centrifuge tube to form 2 separate layers and the samples were centrifuged at 2200 G for 15 min without a brake. After this step, a cloudy buffy coat layer was formed and transferred to a new centrifuge tube and the collected PBMCs were washed with PBS. PBMCs were either frozen in 10% DMSO in FBS solution or taken fresh for DNA isolation. PBMC samples were resuspended in 250 μL SDS lysis buffer (100 mM NaCl, 50 mM Tris–HCl, 5 mM EDTA, 1% SDS), vortexed and sonicated to lyse all cells. After lysis of the cells, they were incubated at 95°C for 5 min and at room temperature on the bench for 5 min, respectively. We added 3–4 μL proteinase K (10 mg/mL) to the samples and incubated at 37°C for 1–2 h. Following incubation, 250 μL phenol chloroform isoamyl alcohol (PCI) was added and mixtures were vortexed vigorously for 30 s. Samples were centrifuged at 13000 G for 10 min and at the end of this spin the upper aqueous layer was transferred to a new 1.5 mL tube without disturbing the lower organic phase. We added 500 μL 100% EtOH and 25 μL 3 M Na-Acetate to the mixture and vortexed. Following centrifugation at 14000 G for 15 min, the supernatant was discarded; the remaining pellet was washed with 500 μL 70% EtOH, centrifuged at 14,000G for 5 min and the supernatant was discarded. Samples were dried at room temperature to ensure that all ethanol was removed. The pellet was dissolved in 100 μL of double-distilled water. Additionally, 1 μL RNase was added to remove any RNA contamination.

### Amplification of APOL1 gene segment by polymerase chain reaction

We first used primers for the PCR technique to amplify the APOL1 gene segment for the SRA domain specifically to include the possible regions of 2 SNPs and 1 deletion region (Primers are shown in Supplementary Table 1). Following DNA isolation, we performed a PCR reaction to amplify the SRA domain, utilizing a total of 10 ng of DNA, 2 μL of primer mix (final concentration of 1 mM primers mix), 10 μL of NEBNext^®^ High-Fidelity 2X PCR Master Mix (New England Biolabs, Ipswich, MA, USA), and 2 μL of DMSO in dH2O (total 20 μL/reaction). We confirmed the size of the PCR product (~280 bp) using Agarose gel electrophoresis.

### Sanger sequencing

Sanger sequencing is used to evaluate DNA products by individually identifying all deoxynucleotide triphosphates; it allows for the investigation of specific sequences for SNPs, mutations, deletions, and mismatches. Amplified products were purified and sent to Eurofins Genomics Laboratories. Sequencing results were evaluated with the Snap Gene Viewer application for MAC (GSL Biotech LLC, San Diego, CA). We also developed a scoring system for each patient using Sanger results; (i) if a singular peak corresponding to the wild-type (WT) was seen at the SNP region, or if there was a full 6-bp sequence present without any perturbation at the G2 location, we assigned a score of 2–0; (ii) if overlapping peaks were noticed at the SNP locations or overlapping nucleotides appeared at or after the deletion region, we assigned a score of 1–1; (iii) if there was a single peak with a modified nucleotide at the SNP location, and a definitive 6-bp deletion on the sanger chromatogram at the G2 location, we assigned a score of 0–2. These scores were used to evaluate the outcome of transplant patients.

### qPCR for the detection of APOL1 variants

Quantitative real-time PCR was conducted to find the ΔCt values between WT and SNP/Del primers. Briefly, we construct WT primers and “SNP/Del primers” for two SNP loci and 6-bp deletion loci. PCR products were run in 6 sets of reactions to detect SNP/Del and WT genes for each patient (Figures 1B,C). Initially, we used “unmodified exact sequence” primers to match WT, SNP, or Del sequences. After finding no expression differences, we used “modified primers” (Supplementary Table 1 and Supplementary Figure 1).

We used the following qPCR setup formula for each reaction: 1 μL PCR product, 10 μL of 2x SYBR Green qPCR master mix (PowerTrack SYBR Green Master Mix, ThermoFischer Scientific, Waltham, MA, USA), 2 μL of a primer mixture (final concentration of 1 mM primer mixture) in dH2O (total 20 μL/wheel). All primer sequences are listed in Supplementary Table 1. We checked both Sanger sequencing and qPCR results to confirm that both scores were similar.

### Statistical analysis

Statistical analysis was carried out using Graph Pad Prism software. Kidney function results are presented as mean ± standard deviation (SD). To compare patient groups with 0, 1, and 2 risk alleles, we conducted one-way ANOVA tests. Additionally, we employed repeated-measures ANOVA to compare follow-up data within each risk allele category. Statistical significance was set at *p* < 0.05.

## Results

### Evolving strategies in APOL1 variant classification: a haplotype-centric model

**Figure 2:**
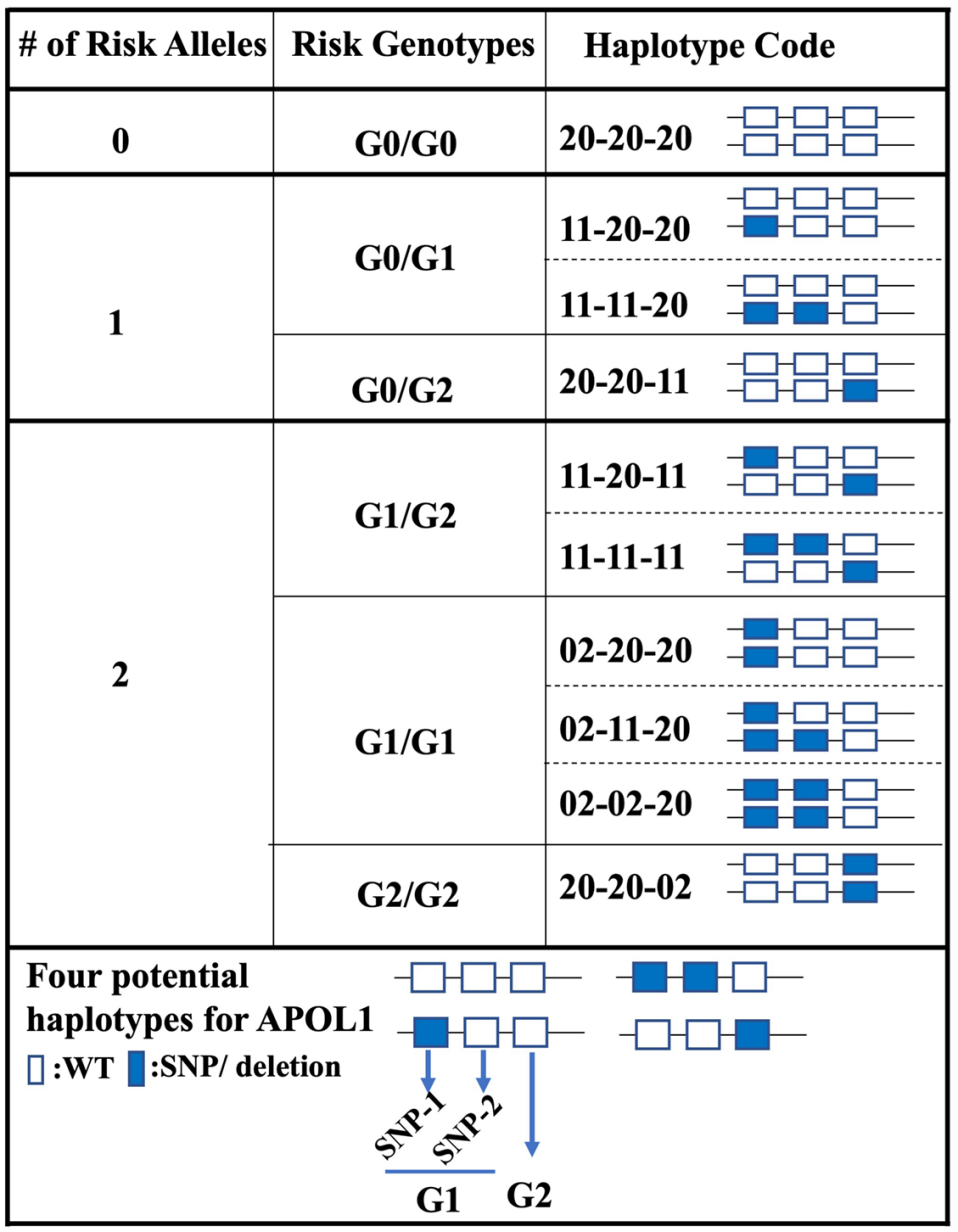
Haplotype-centric classification approach for APOL1 variant status. This figure illustrates the proposed classification model for patients based on their APOL1 variant status. Three primary risk categories (0-risk, 1-risk, 2-risk) are further divided into a total of 10 groups using a novel 6-digit scoring system focused on APOL1 haplotypes.

The stratification of patients based on their APOL1 variant status is critical for the prediction of the clinical course of patients in APOL1-related research, particularly in large cohort studies like APOLLO (15). Current classification systems generally categorize patients into three groups, depending on the number of risk alleles (0-risk, 1-risk, 2-risk), or into six groups, when the genotypes of individual alleles are considered (G0/G0 for 0-risk, G0/G1 and G0/G2 for 1-risk, and G1/G2, G1/G1, and G2/G2 for 2-risk alleles). Here, we introduce a novel classification approach that considers the haplotypes inherited from each parent and potential combinations. In this new framework, we used a scoring system that focused on two specific SNP locations and the deletion locus. This offers a more nuanced way to classify APOL1 variant status, which could lead to robust studies and a possible estimation of patient progression after transplantation. Considering the equal inheritance of genetic material from each parent, we propose the possibility of attributing a scoring mechanism to individual SNPs and deletions. In this proposed model, a score of 2–0 was given when both DNA sequences carried the WT allele. In contrast, a score of 1–1 was given when one DNA sequence displayed the WT allele and the other had a SNP or deletion. Finally, a score of 0–2 was assigned when both DNA sequences carried a SNP or deletion. We assigned this scoring for each SNP and deletion so as to get a 6-digit code for each patient. For instance, a score of 20–20-20 would represent a WT status for both SNPs and deletions, corresponding to the genotype G0/G0. With this new coding system, we obtained 10 different groups (1 for the 0-risk, 3 for the 1-risk, and 6 for the 2-risk allele, Figure 2).

### Demographic characterization of donors and stratification of APOL1 genetic variants by sanger sequencing

**Figure 3:**
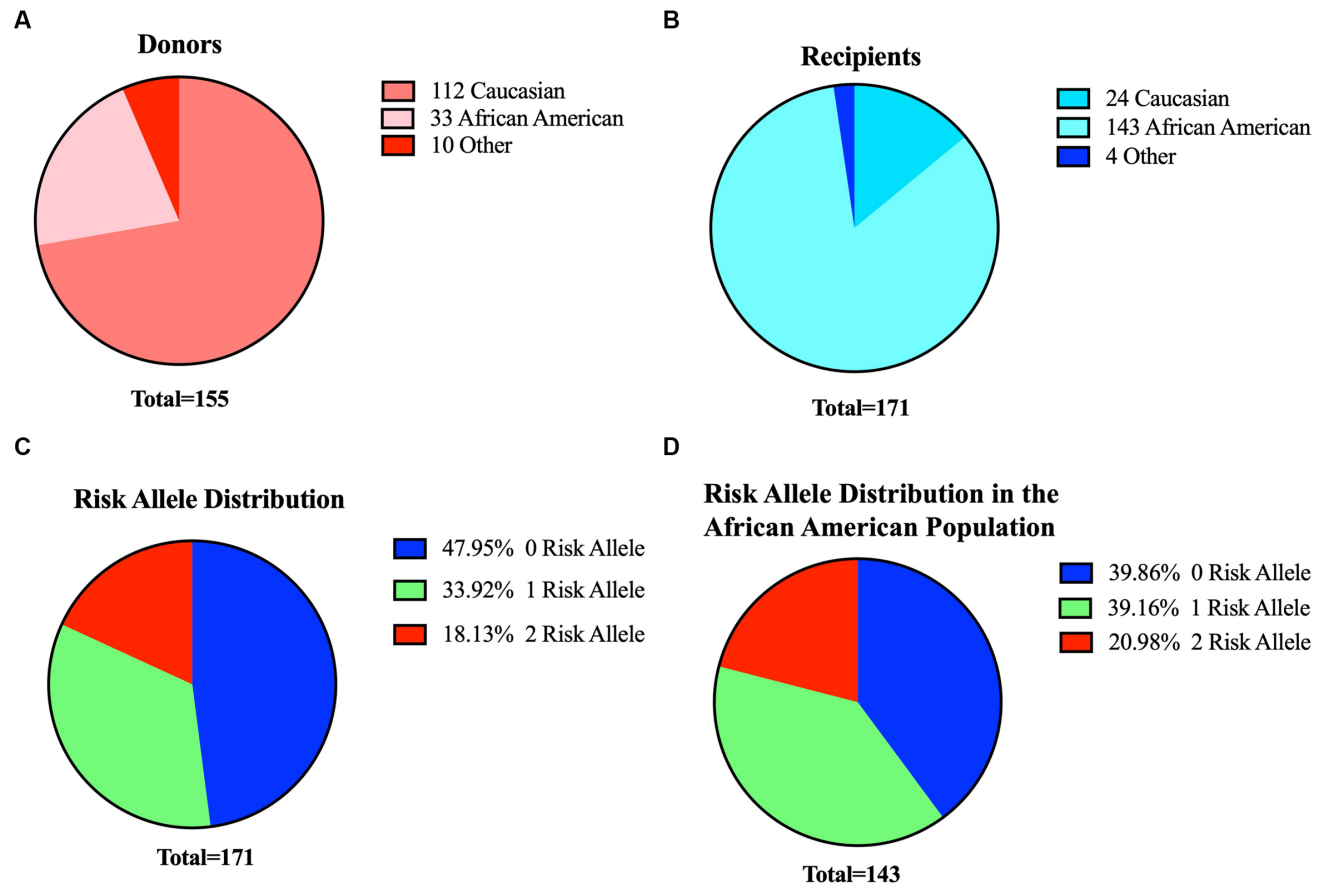
Profiles of donors and recipients in our cohort and overview of risk metrics for recipients. Distribution of donors **(A)** and recipients **(B)** based on their ethnicity. **(C)** Distribution of APOL1 risk variants among the 171 recipients in our study **(D)**. Risk allele distribution for African-American recipients only (n:143) in the cohort. Blue:0-risk, green:1-risk, red:2-risk.

In our kidney transplant cohort, we obtained 155 donor organs (16 paired, 139 individual kidney transplants) and 171 recipients. Within the donor group, 112 individuals identified as Caucasian, 33 as African-American, and 10 were of other racial backgrounds. On the other hand, the recipient cohort included 143 African Americans, 24 Caucasians, and 4 individuals from other racial backgrounds (Figures 3A,B and Table 1). Given the higher proportion of African-American patients in our recipient cohort, we anticipated a higher prevalence of APOL1 risk variants. By analyzing the distinct peaks observed in the Sanger chromatograms, we successfully ascertained the genotype at each respective position for SNPs and deletions (Supplementary Figure 2). Sanger sequencing results revealed that 82 recipients (48%) had no risk allele (G0/G0), while 58 recipients (34%) had one risk allele and 31 recipients (18%) had two risk alleles.

**Table 1:** APOL1 haplotype codes and patient ethnicities.

Haplotype code	Risk genotype	Total	Caucasian	AA	Other
202020 0 Risk Allele	G0/G0	82	23	57	2
111120	G0/G1	37 (64%)	1	36 (64.3%)	0
112020	G0/G1	3 (5%)	0	2 (3.7%)	1
202011	G0/G2	18 (31%)	0	18 (32%)	0
1 Risk Allele		58	1	56	1
020220	G1/G1	15 (48.4%)	0	14 (46.6%)	1
202002	G2/G2	4 (13%)	0	4 (13.3%)	0
111111	G1/G2	10 (32.2%)	0	10 (33.3)	0
21120	G1/G2	2 (6.4%)	0	2 (6.6%)	0
2 Risk alleles		31	0	30	1
	Total	171	24	143	4

We further stratified our patients based on both their APOL1 risk genotype and APOL1 haplotype codes. In this cohort, we observed the presence of all three possible haplotype codes associated with a 1-risk allele, with the 11-11-20 haplotype manifesting at the highest frequency (64%). Similarly, only four of the six putative haplotypes were detected in recipients with two-risk alleles. The haplotype 02–02-20 had the highest prevalence (48.4%). Notably, haplotypes 02–20-20 and 11-20-11 were absent in our data set due to the limited number of patients in the cohort. In our recipient cohort, one individual of Caucasian descent and one individual of a different racial background each carried one risk allele, while another individual of a different racial background carried two risk alleles. Within the African-American subset of the cohort, 57 (40%) of recipients had no risk allele, 56 (39%) had one risk allele, and 30 (21%) had two risk alleles (Figures 3C,D and Table 1).

### Probe-independent quantitative PCR as a robust alternative to genetic testing and sanger sequencing for variant identification

#### SNP1 (rs73885319) region and SNP-2 (rs60910145) region

**Figure 4:**
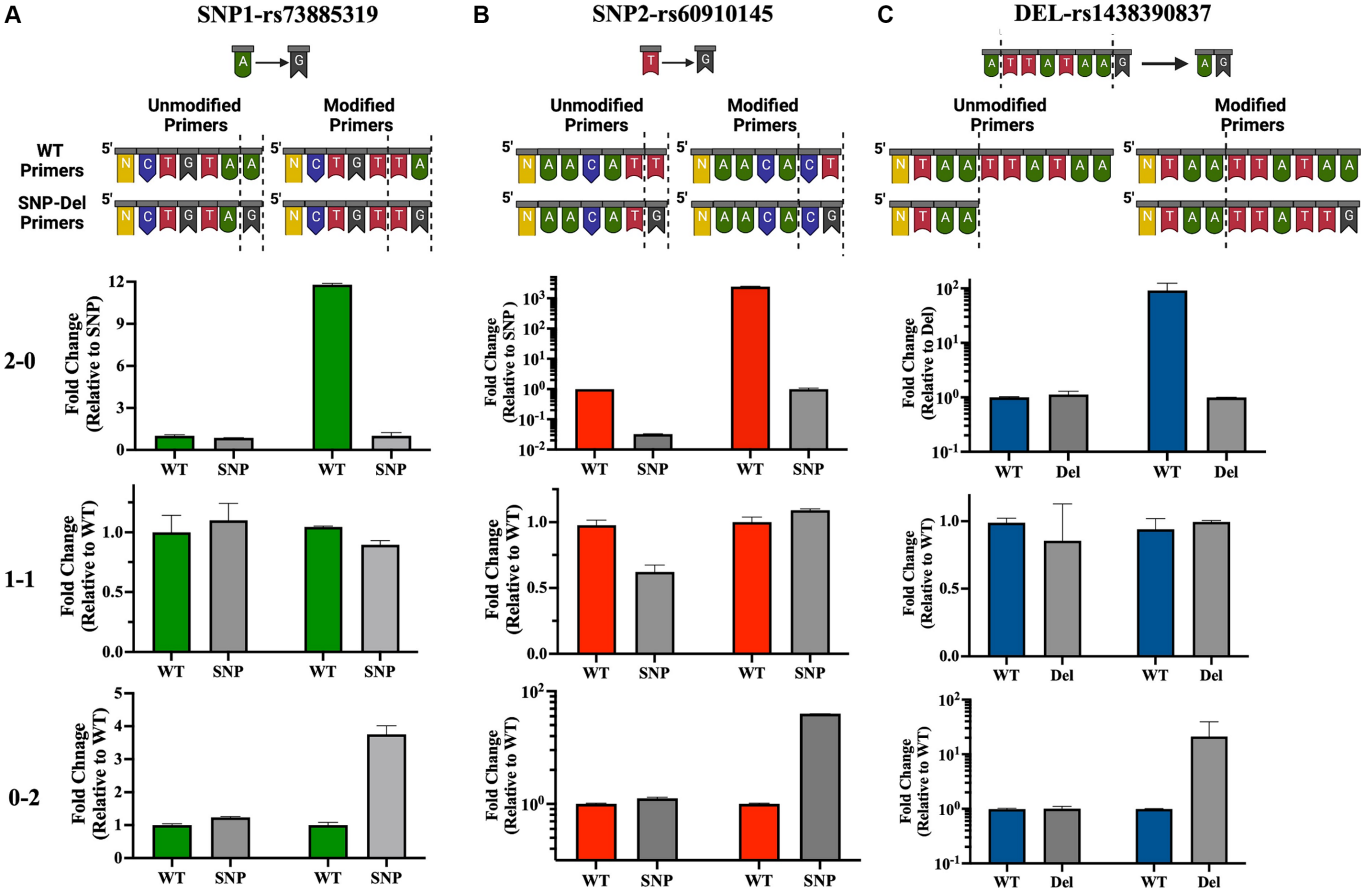
Development and validation of allele-specific qPCR primers for APOL1 variant detection. Top panel: Illustration of standard “unmodified” or custom-designed “modified” primer sequences for the SNP-1 **(A)**, SNP-2 **(B)** and deletion **(C)** loci. Altered or deleted base pairs are demarcated by dotted lines. Bottom panel: Quantitative analysis of qPCR data illustrates homozygous or heterozygous variants. 2–0: homozygous WT, 1–1: heterozygous, 0–2: homozygous SNP/deletion (N: number of bases remaining in the primer sequence). This figure has been partially generated with Biorender program.

Following the initial identification of APOL1 variants by clinical genetic testing and Sanger sequencing, we selected a subset of patients with pre-identified genotypes to serve as controls. Our initial approach involved designing allele-specific primers to distinguish the WT allele from the SNP allele. This design was predicated on the concept that a single nucleotide difference at the 3′ end of primer oligos could significantly alter the PCR efficiency. Our objective was to detect this efficiency difference in real-time quantitative PCR as a ΔCt value, thereby eliminating the need for an internal probe (Figure 1C). We termed this set of primers “unmodified primers,” which differed only by a single base at the SNP locus. Specifically, for the SNP1 (rs73885319) region, we implemented a single base change at the end of the sequences (A → G), and for the SNP-2 (rs60910145) region, we used a single base change at the end of the sequences (T → G). Initial qPCR assays using these primers yielded similar expression levels for both WT and SNP genotypes, as illustrated in Figures 4A,B (left part of each section). It became evident that a single nucleotide change at the 3’end of the primers was not sufficient to distinguish between the groups by qPCR. Therefore, we opted to modify the primers by introducing a second base pair alteration adjacent to the SNP location to influence the binding efficiency of the primers to their target regions. In this modified format, we changed the second base from a purine to a pyrimidine pair for SNP1 or kept it as a pyrimidine but changed to a cytosine for SNP2, thereby increasing our ability to detect differences between the WT and SNP genotypes. So, for the SNP1 region, we changed the last two nucleotides from AA to TA for the WT primer and from AG to TG for the SNP primer. Similarly, for the SNP2 region, we changed the last two nucleotides from TT to CT for the WT primer and TG to CG for the SNP primer. We called these primer pairs “modified primers” (Figures 4A,B and Supplementary Figures 1, 3).

**Figure 5:**
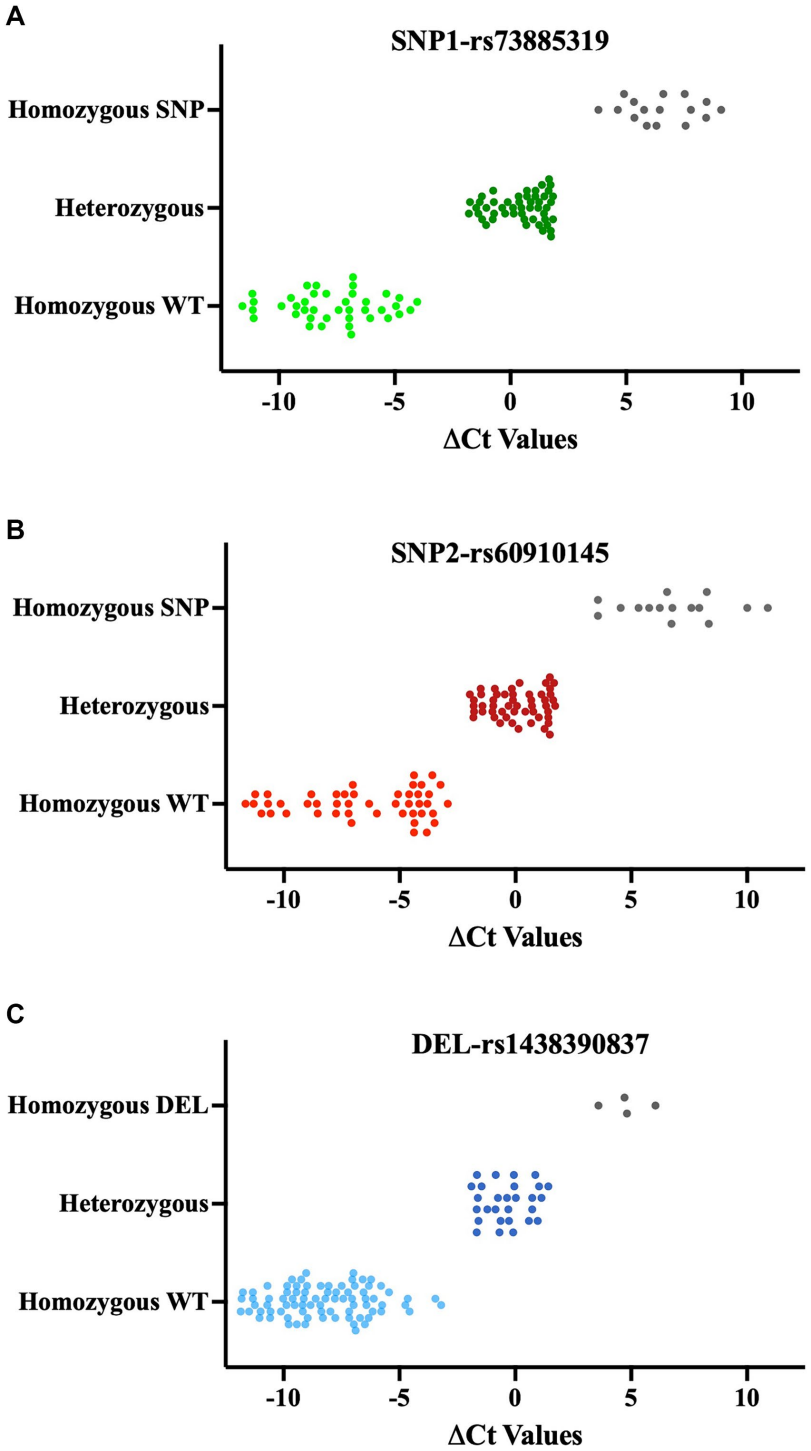
ΔCt values of QPCR results for SNPs **(A, B)** and deletions **(C)**. qPCR results of each SNP and deletion are classified based on Sanger Sequencing results. Each data point shows the mean of ΔCts values (ΔCt = Ct value with modified WT primer- Ct value with modified SNPs/Del primer). Lighter color: Homozygous WT, Darker color: Heterozygous, Gray: Homozygous SNP or Deletion.

In this new qPCR setting, we observed at least a 4-fold differential expression (ΔCt ≥ 2) between the WT primers and SNP primers when using homozygous WT or homozygous SNP genomic DNA for both SNP loci. In qPCR where we used heterozygous DNA, the differences in expression levels between the two primers were uniform and did not exceed a 4-fold difference (Figures 4A,B, right side in each panel, Figures 5A,B, and Supplementary Figures 1, 3). Upon optimizing our primers for specific SNP detection, we proceeded to evaluate all recipient genotypes to determine ΔCt values, which were then utilized to calculate the threshold value. As shown in Figures 5A,B, a distinct separation in ΔCt values was evident among all three genotype groups.

#### Deletion of the rs143830837 region

Our PCR reaction was initially configured using a common forward primer, along with reverse primers with exact matches designed to detect the presence or absence of the 6-bp deletion identified as “TTATAA” (these primers were referred to as “Unmodified Primers”). In this quantitative PCR setup, we did not observe any notable expression differences between patients with WT DNA and those with 6-bp deletions in their DNA (Figure 4C, left part in each section, Supplementary Figures 1, 3). Drawing on our experience with SNP primers, where we distinguished between wild-type and SNP DNA by modifying 2 base pairs at the 3′ end of the primer, we applied the same approach to the deletion region. We chose to extend the primers to create a 2-base pair mismatch between them. However, within the rs143830837 Region, there is a repetitive sequence of adenine and thymine bases (5′-ATAA). We opted to extend our WT primers by incorporating the exact sequence from the 3′ end. For the Del primers, we used the same template as the WT primer and extended it with the exact sequences of the DNA containing the deletion, resulting in 2 base differences (ending with “AA” in the WT primer and “TG” in the Del primer). These modified primers were referred to as “Modified Primers.”

In this qPCR setup, we observed expression differences of at least 4-fold for the WT genotype compared to the deletion genotype in the 0–2 and 2–0 genotypings. In the 1–1 genotyping, the differences in expression levels between the two primers were equal and less than 4-fold (Figure 4C, right part of each section, Figure 5C, Supplementary Figure 1). The original quantitative PCR graphs for the SNP loci and the deletion locus are shown in Supplementary Figure 3.

We validated our qPCR results by Sanger sequencing for our patients. We have summarized the ΔCt values along with their corresponding Sanger histograms for 20 patients (more than 10%) in Supplementary Figure 4.

### APOL1 variants show no early impact on renal function metrics in kidney transplant recipients

**Figure 6:**
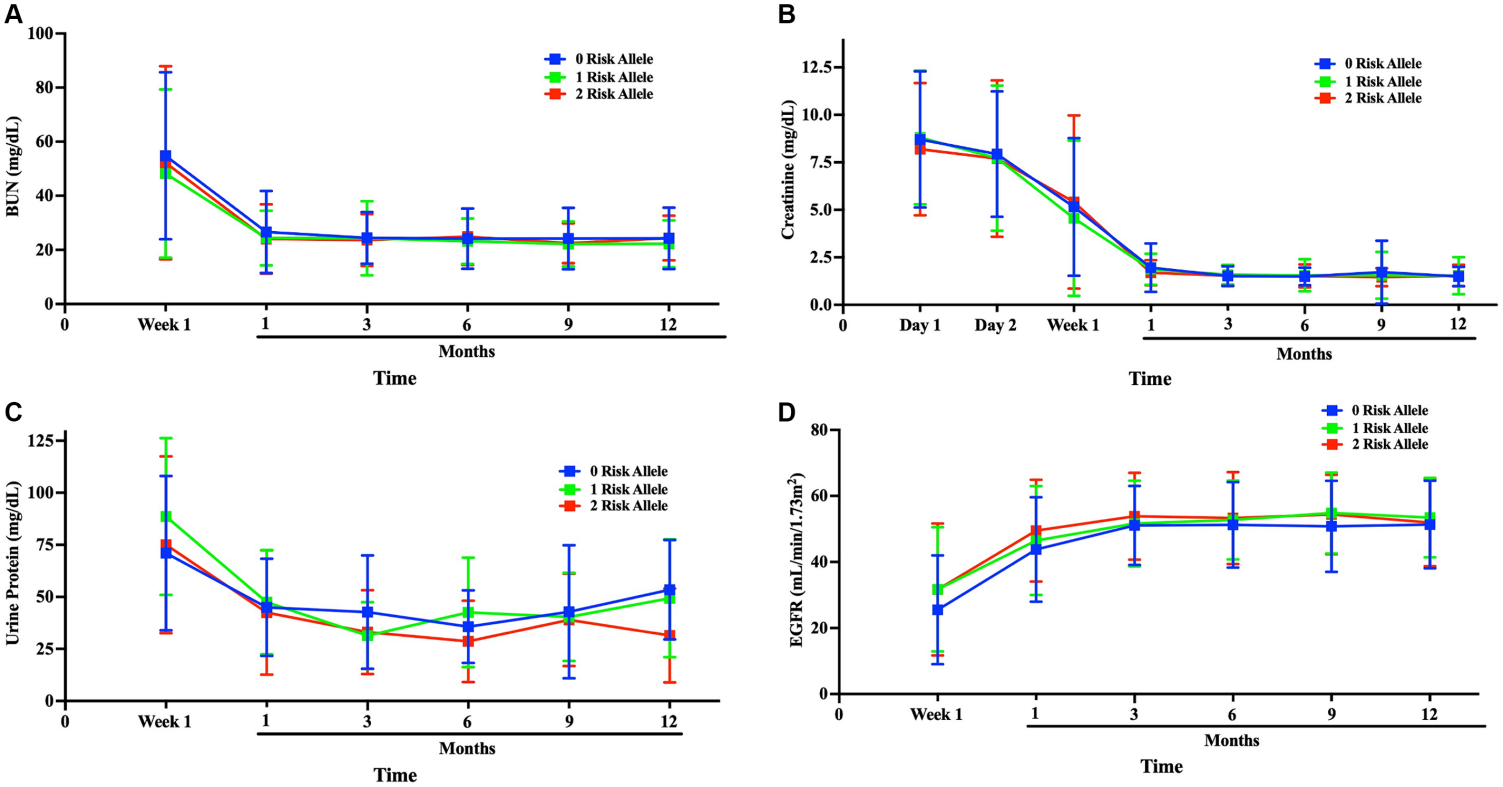
Comparison of kidney function among APOL1 risk alleles after transplantation kidney function was compared after transplantation to up to 1-year follow-up; **(A)** blood BUN, **(B)** Creatinine, **(C)** Urine protein, **(D)** eGFR progression Blue:0-risk, green:1-risk, red:2-risk.

As part of the pre- and post-operative evaluation of patients at days 1 and 2, week 1, and months 1, 3, 6, 9 and 12 to monitor their recovery progress, the levels of Blood Urea Nitrogen (BUN), creatinine, urine protein, and Estimated Glomerular Filtration Rate (eGFR) were measured to assess the functionality of the transplanted kidneys. We categorized the results according to the presence or absence of risk alleles. Over the course of the first month, BUN, creatinine, and urine protein levels showed a significant decrease before stabilizing at 3-, 6-, 9- and 12-month follow-ups (*p* < 0.05). Similarly, eGFR levels showed a progressive improvement during the first month and then remained stable at the 3-, 6-, 9- and 12-month follow-ups (*p* < 0.05). Kidney function was similar and improved in all risk allele groups and remained stable at the 3-month follow-up. Importantly, no notable differences were observed in these renal function indicators when stratifying the recipients based on APOL1 risk allele status (Figure 6 and Supplementary Figure 5).

## Discussion

This study focused on APOL1 variants, their identification, patient genotype classification, haplotype coding, and implications for kidney transplant recipients. We introduced a novel haplotype-centric model for APOL1 variant classification and employed quantitative PCR as an alternative genotyping method. Furthermore, we analyzed early postoperative renal function metrics in kidney transplant recipients based on their APOL1 risk allele status. These findings provided valuable insights into the expanding field of APOL1 and kidney disease research.

Our innovative haplotype-centric model marks a significant advance over traditional APOL1 classification methods, which have been limited to a few risk allele categories or genotypes. Our approach introduces a unique 6-digit code that allows for a wide range of haplotype combinations for a more nuanced understanding of APOL1-related risks. Within our patient group, we successfully identified 8 out of 10 possible haplotype combinations, with the remaining two eluding us, likely due to our cohort’s limited size.

Several methods are currently being employed to identify APOL1 risk variants in individuals, including the measurement of APOL1 variants bound to HDL particles. APOL1, a structural protein of HDL, is cleared from the bloodstream by the kidneys. Assessing the quantity and quality of mutant APOL1-HDL proteins may aid in disease estimation. In a recent study involving 3,450 individuals, liquid chromatography-mass spectrometry (LC–MS) was used to measure plasma APOL1 variant levels, but no association with kidney function was found. The study concluded that circulating APOL1 levels may not correlate with mutant APOL1-HDL protein forms (15). Additionally, another group identified APOL1 gene variants with blood and serum measurements using LC–MS, analyzing surrogate peptides to identify different gene variants (16).

The probe-based method is another way to identify APOL1 gene variants. It involves isolating DNA from blood samples and using fluorescently labeled detection probes along with PCR and a 5′-nuclease assay. When a probe matches the target DNA containing APOL1 variants, it is degraded by 5′-nuclease polymerase activity, releasing the reporter dye and producing a detectable fluorescent signal. The TaqMan SNP Genotyping Assay, commonly used in clinical settings, is a standard choice for this method (17).

Our study utilized custom-designed primers and quantitative PCR for APOL1 gene variant identification, offering quicker and more cost-effective results. We extracted DNA from whole blood and then used variant-specific primers to quantify threshold cycle (Ct) values. Validation by Sanger sequencing yielded a 100% success rate, making this method accessible to standard research laboratories.

Identifying APOL1 gene variants in potential kidney donors and transplant recipients holds crucial health implications. For donors, possessing these variants increases the risk of kidney dysfunction and end-stage renal disease, potentially making kidney donation a risky endeavor. Moreover, it can impact recipients, leading to early allograft dysfunction and rejection. A study by Reeves-Daniel et al. revealed significantly lower graft survival rates in donors with 2 APOL1 gene variants compared to those with 1 variant (50% vs. 75% over 3.5 years). Notably, having 2 APOL1 variants conferred a higher risk (HR-3.84) than HLA mismatch and cold ischemia time (HR-1.52 and HR-1.06, respectively) (18).

When considering the genotype of transplant patients, in a 5-year retrospective study of 119 African-American transplant recipients, those with 2 APOL1 gene variants had a similar allograft survival rate to those with 1 variant (approximately 50% survival for both groups), regardless of donor genotype. Despite their increased risk of native kidney disease, allograft outcomes remained comparable (19). A recent research by Zhang et al. presented conflicting results. In the Genomics of Chronic Allograft Rejection (GOCAR) study, patients with 0 APOL1 risk allele had higher transplant survival rates than those with 1 allele, and 1 allele carriers had better survival than those with 2 alleles during 7 years of follow-up after transplantation. The Clinical Trials in Organ Transplantation (CTOT) study showed a similar pattern of survival curves, though the within-group differences were not statistically significant at 5-year follow-up (10). Our cohort consisted of recent transplant recipients, meaning we do not yet have data on long-term outcomes. However, the APOL1 status of recipients did not significantly affect kidney function in the first year after transplantation. It is worth noting that tacrolimus, a widely used medication, can have nephrotoxic effects and directly influence serum creatinine levels (20). Nonetheless, since all of our patients were administered tacrolimus, this should not bias our analysis.

The short post-transplant follow-up period of our study limited our ability to observe long-term effects and draw definitive conclusions about the impact of APOL1 variants on kidney transplant outcomes. While our study comprised 171 primarily African-American patients, and revealed a high percentage of APOL1 variants (18% with 2 Risk Alleles and 34% with 1 Risk Allele) among transplant patients, the findings may not fully represent all kidney transplant populations, as the observed high percentage of APOL1 variants may be influenced by the demographics of our cohort and may not be universally applicable. Due to study design constraints, we could not fully compare all haplotype risk codes as intended, potentially limiting the depth of our analysis and the generalizability of our findings. Our assessment of recipient APOL1 status and its impact on kidney function was restricted to one-year post-transplant outcomes, necessitating longer-term follow-up to evaluate the durability of kidney function, and the persistence of observed effects. While our qPCR-based APOL1 genotyping method showed promise, further validation in larger multicenter studies is needed to confirm its reliability and accuracy in diverse populations. Although our study proposes a new haplotype-centric classification for APOL1 variants, its utility and applicability require validation in larger cohorts, like the APOLLO study (15), to ensure its effectiveness as an alternative categorization method.

Our study’s reliance on DNA samples extracted from blood was limited by potential variability in sample quality, which could introduce bias into the genetic analysis.

### Achievements

Our probe-independent qPCR method effectively detects APOL1 gene variants. It enabled us to extract DNA from minimal blood samples and conduct APOL1 genotyping using qPCR within a total time of 3–4 h. This breakthrough provides a new opportunity for identifying APOL1 gene variants in both clinical and basic research.

## Data availability statement

The original contributions presented in the study are included in the article/Supplementary material, further inquiries can be directed to the corresponding authors.

## Ethics statement

The studies involving humans were approved by the local ethics committee by The Institutional Review Board of the University of Tennessee Health Science Center (IRB Approval Number: 20-07838-XP). The studies were conducted in accordance with the local legislation and institutional requirements. The participants provided their written informed consent to participate in this study.

## Author contributions

MD: Conceptualization, Data curation, Formal analysis, Investigation, Methodology, Validation, Visualization, Writing – original draft, Writing – review & editing, Resources. CW: Data curation, Methodology, Resources, Writing – review & editing. HI: Investigation, Methodology, Resources, Writing – review & editing. NM: Formal analysis, Investigation, Methodology, Validation, Writing – review & editing. GR: Investigation, Methodology, Resources, Validation, Writing – review & editing. EG: Methodology, Resources, Writing – review & editing. JE: Funding acquisition, Investigation, Project administration, Resources, Supervision, Writing – review & editing. ABh: Resources, Writing – review & editing. MT: Resources, Writing – review & editing. NN: Resources, Writing – review & editing. CE: Resources, Writing – review & editing. RH: Resources, Writing – review & editing. JV: Funding acquisition, Resources, Supervision, Writing – review & editing. ABa: Investigation, Methodology, Supervision, Writing – review & editing, Conceptualization, Visualization. CaK: Funding acquisition, Investigation, Methodology, Project administration, Resources, Supervision, Validation, Writing – original draft, Writing – review & editing, Conceptualization. CeK: Conceptualization, Data curation, Formal analysis, Funding acquisition, Investigation, Methodology, Project administration, Resources, Supervision, Validation, Visualization, Writing – original draft, Writing – review & editing.
