# Supplementary material for: Unveiling APOL1 haplotypes in a predominantly African-American cohort of kidney transplant patients: a novel classification using probe-independent quantitative real-time PCR
Source: Front Med (Lausanne). 2024 Apr 10;11:1325128. doi: 10.3389/fmed.2024.1325128 (PMC11039853; doi:10.3389/fmed.2024.1325128)
Supplement: Supplementary file 1 [file Data_Sheet_2.PDF]

Supplementary Table 1

List of APOL1 Variant QPCR Primers

**SRA Domain Primers**	
SRA-Forward	AATCTAGAGACCGGGATTACCAGCAGTA
SRA-Reverse	TTCCTGCCTTCTCCTTGCTG
**SNP1(**rs73885319)	
**Unmodified (Exact Sequence) Primers:**	
rs73885319-WT Forward (A)	CACGGATGTGGCCCCTGTA**A**
rs73885319-SNP1 Forward (G)	CACGGATGTGGCCCCTGTA**G**
Reverse(common)	GCCCTGTGGTCACAGTTCTT
**Modified (2 Mismatch) Primers**	
rs73885319-WT Forward: (TA)	CACGGATGTGGCCCCTGT**TA**
rs73885319-SNP1 Forward: (TG)	CACGGATGTGGCCCCTGT**TG**
Reverse (common)	GCCCTGTGGTCACAGTTCTT
**SNP2** (rs60910145)	
**Unmodified (Exact Sequence) Primers:**	
rs60910145-WT Forward (T)	AGCTGGAGGAGAAGCTAAACAT**T**
rs60910145-SNP2 Forward (G)	AGCTGGAGGAGAAGCTAAACAT**G**
Reverse (common)	GCCCTGTGGTCACAGTTCTT
**Modified (2 Mismatch) Primers**	
rs60910145-WT Forward (AT)	AGCTGGAGGAGAAGCTAAACA**CT**
rs60910145-SNP2 Forward (AG)	AGCTGGAGGAGAAGCTAAACA**CG**
Reverse (common)	GCCCTGTGGTCACAGTTCTT
**Deletion RS 143830837 Region**	
**Unmodified (Exact Sequence) Primers**	
rs143830837-Forward (common)	AGCTGAGGAGCTGAAGAAGG
rs143830837-WT Reverse (TTATAA)	GGTCCGCCTGCAGAATC**TTATAA**
rs143830837-Del Reverse (-)	GGTCCGCCTGCAGAATC
**Modified (2 Mismatch) Primers**	
rs143830837-Forward (common)	AGCTGAGGAGCTGAAGAAGG
rs143830837-WT Reverse (AA)	TTGGTCCGCCTGCAGAATCTTAT**AA**
rs143830837-Del Reverse (TG)	TTGGTCCGCCTGCAGAATCTTAT**TG**

Supplementary Figure 1

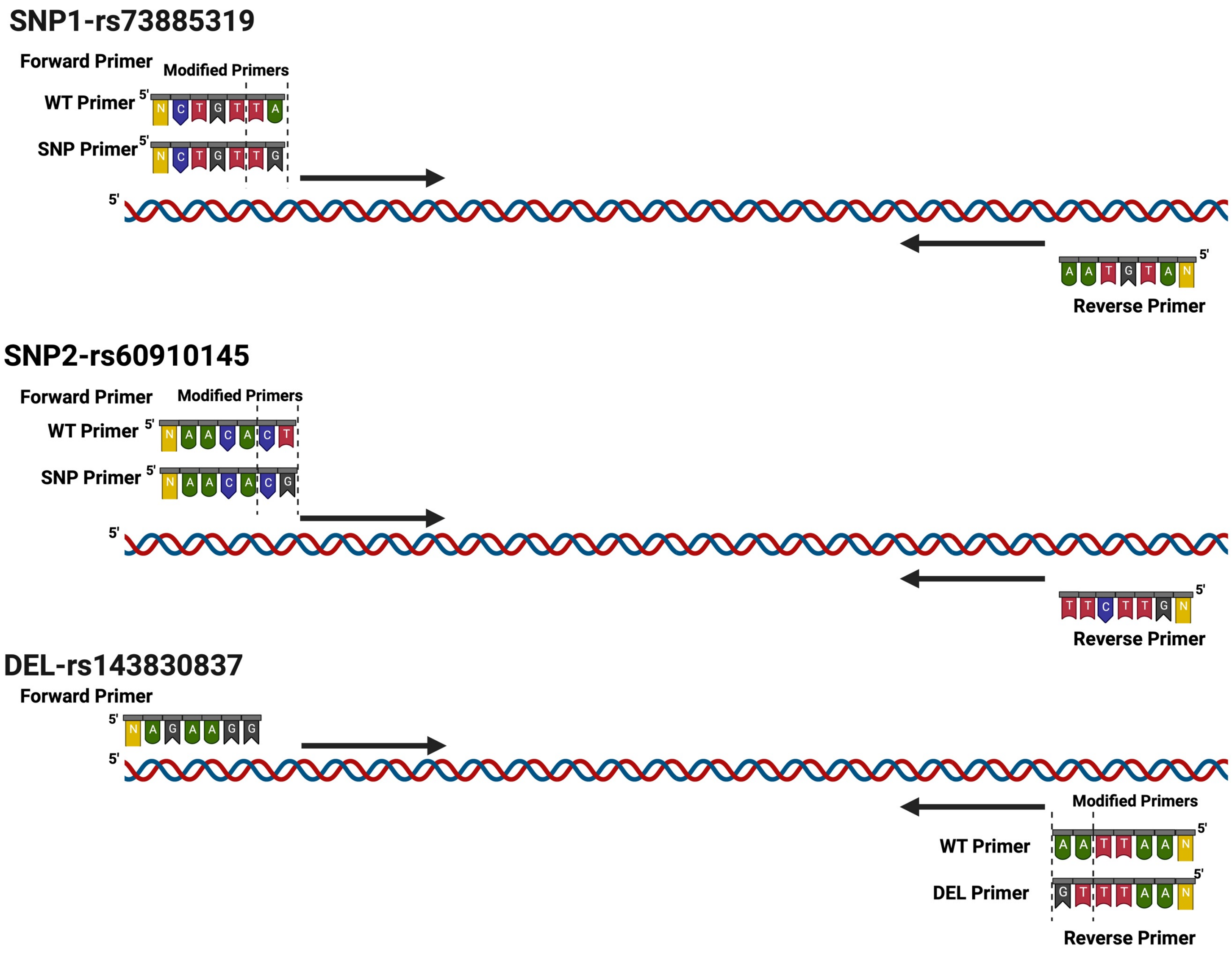

Supplementary Figure 1 Our primers design for detection of APOL1 gene variants (N: Remaining bases in the primer sequence)

Supplementary Figure 2

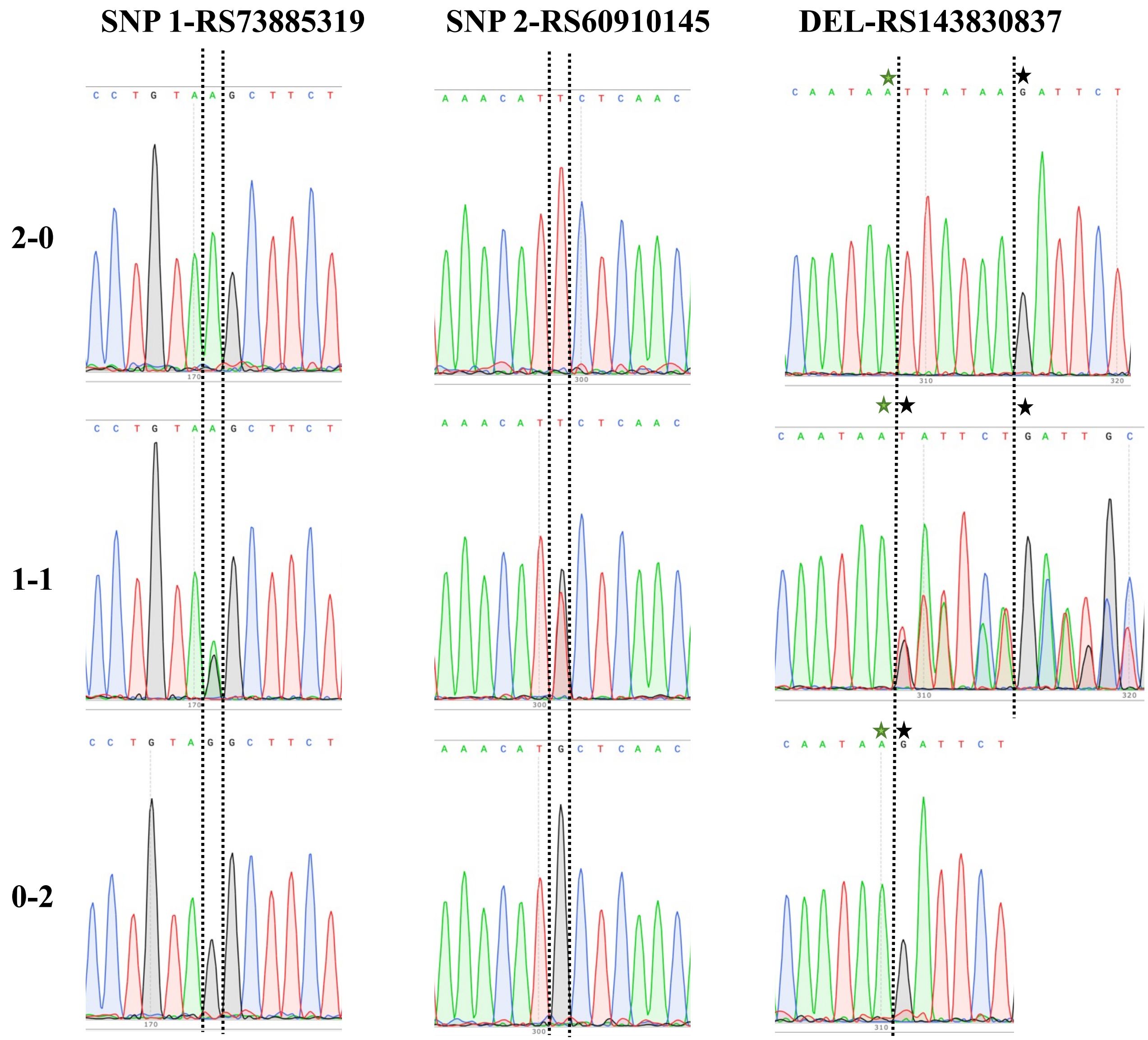

Supplementary Figure 2 All possible genotypes were determined with Sanger Sequencing for each SNPs and deletion.

Supplementary Figure 3

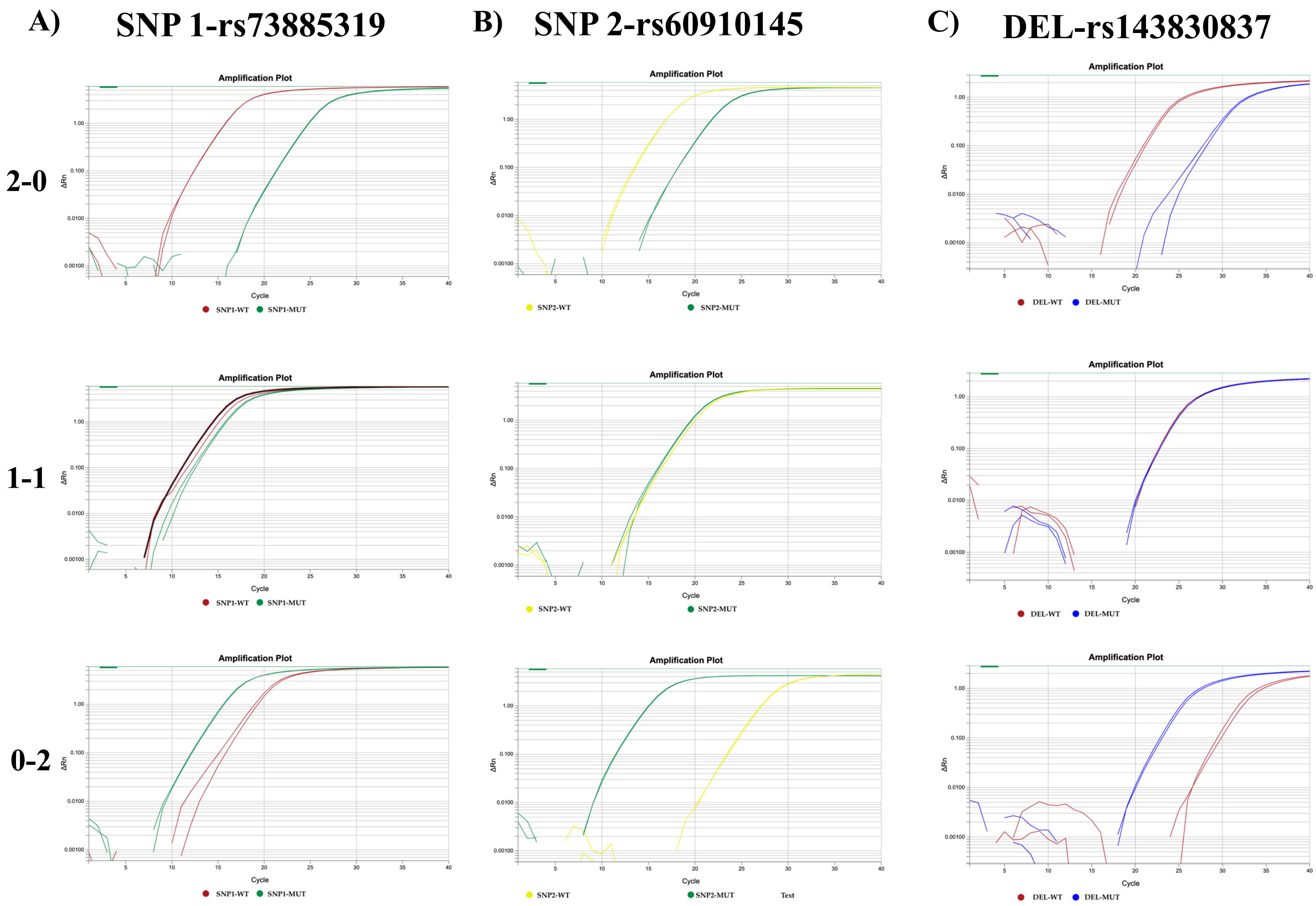

Supplementary Figure 3 Raw Ct graphs were shown to confirm that modified primers can distinguish all possible genotypes.

Supplementary Figure 4 (1/2)

	rs73885319		rs60910145		rs143830837	
	ΔCt	Sanger-Histogram	ΔCt	Sanger-Histogram	ΔCt	Sanger-Histogram
**Patient-1** G0/G0(20-20-20)	-5.35	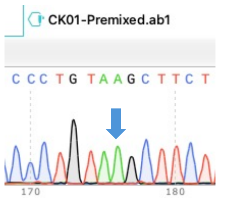	-11.2	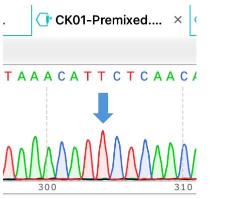	-9.02	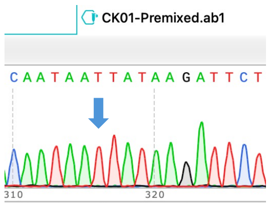
**Patient-2** G1/G1(02-02-20)	4.38	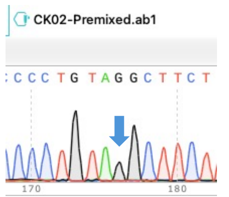	3.46	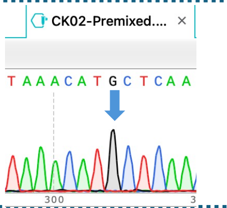	-8.08	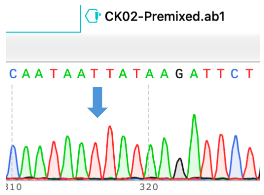
**Patient-3** G0/G0(20-20-20)	-9.07	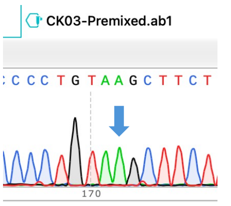	-8.41	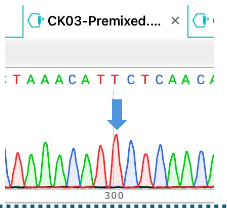	-6.41	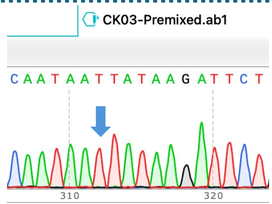
**Patient-4** G0/G0(20-20-20)	-8.38	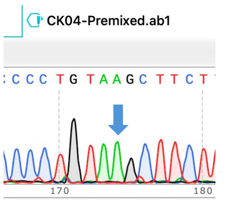	-4.05	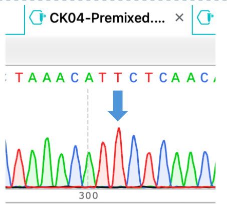	-8.61	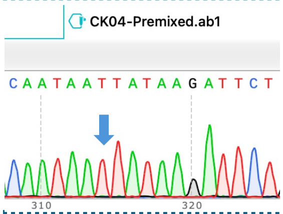
**Patient-5** G0/G1(11-11-20)	-0.89	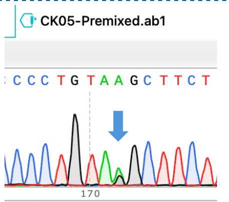	-1.43	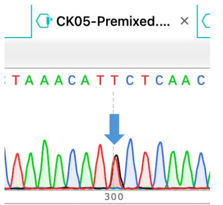	-8.19	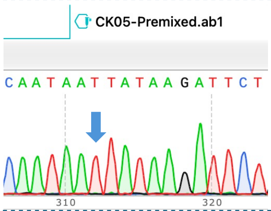
**Patient-6** G0/G1(11-11-20)	1.73	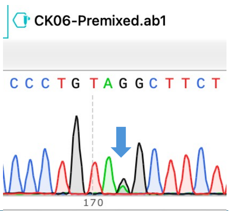	-1.48	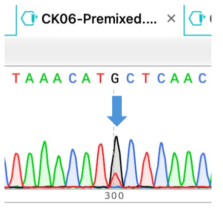	-8.05	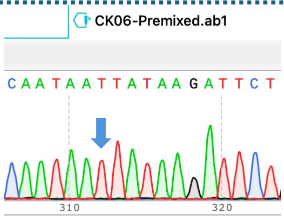
**Patient-7** G0/G1(11-11-20)	-0.35	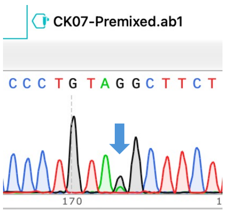	-0.45	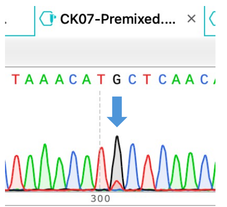	-7.97	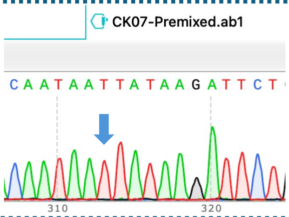
**Patient-8** G0/G2(20-20-11)	-8.68	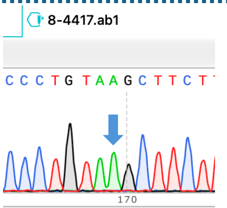	-10.97	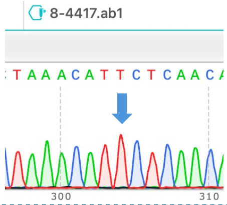	-1.29	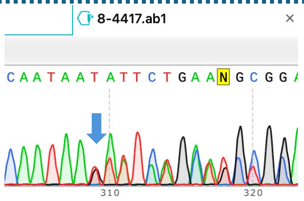
**Patient-9** G0/G1(11-11-20)	-0.85	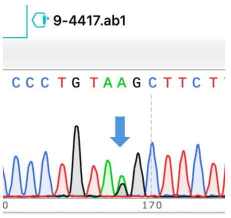	-0.15	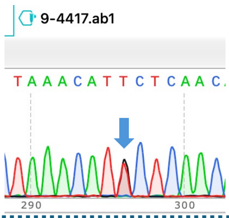	-7.78	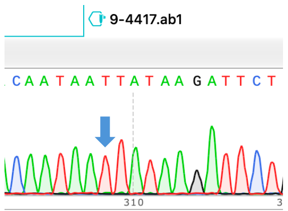
**Patient-11** G1/G1(02-02-20)	5.35	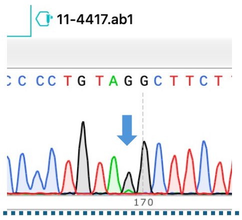	4.54	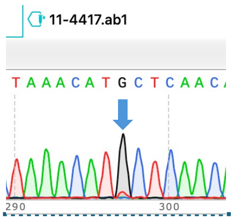	-6.15	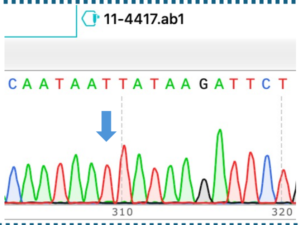

Supplementary Figure 4 (2/2)

	rs73885319		rs60910145		rs143830837	
	ΔCt	Sanger-Histogram	ΔCt	Sanger-Histogram	ΔCt	Sanger-Histogram
**Patient-14** G1/G1(02-02-20)	4.38	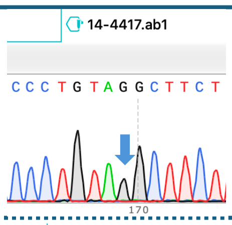	3.46	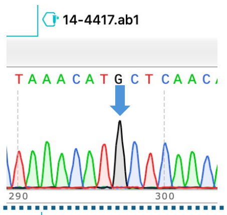	-8.08	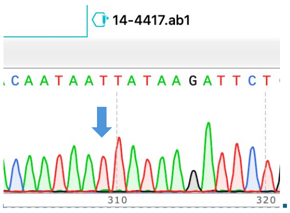
**Patient-21** G1/G1(02-02-20)	5.33	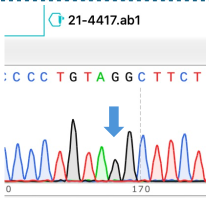	5.77	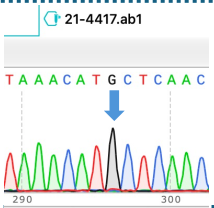	-6.22	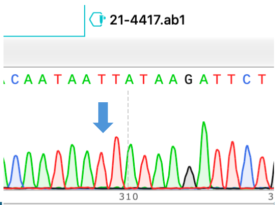
**Patient-23** G1/G2(11-11-11)	-1.748	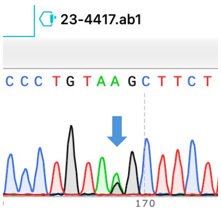	-0.99	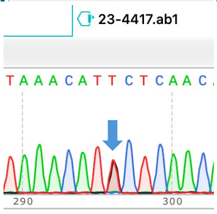	-1.65	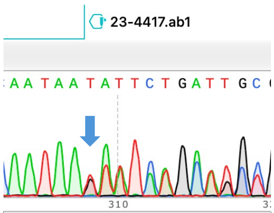
**Patient-25** G0/G1(11-11-20)	1.39	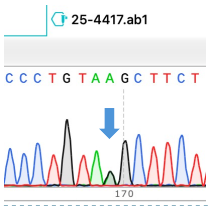	1.48	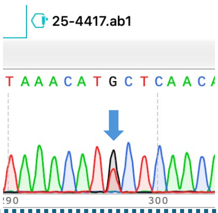	-6.58	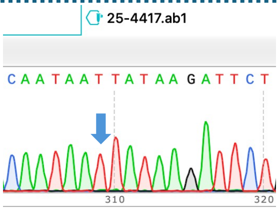
**Patient-28** G1/G1(02-02-20)	7.79	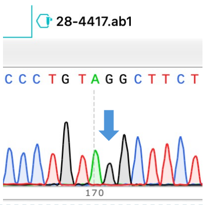	8.25	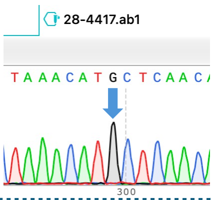	-7.03	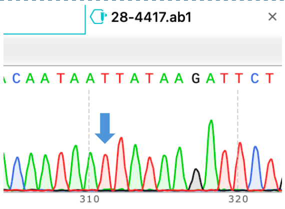
**Patient-29** G0/G2(20-20-11)	-5.88	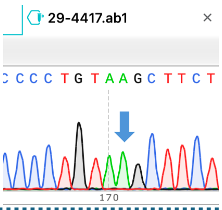	-4.41	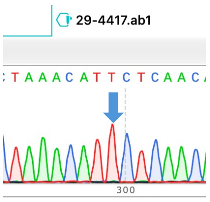	-1.16	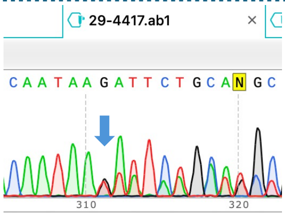
**Patient-31** G1/G1(02-02-20)	9.09	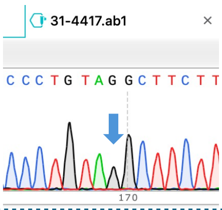	8.34	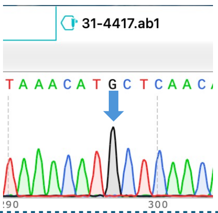	-6.48	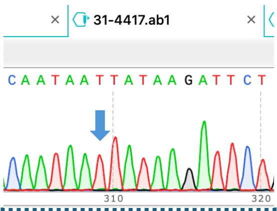
**Patient-35** G0/G1(11-20-20)	-1.08	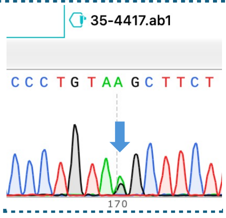	-6.70	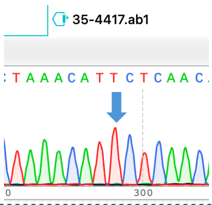	-6.32	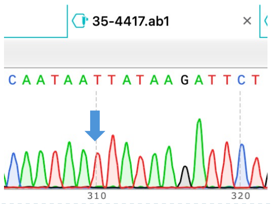
**Patient-54** G0/G2(20-20-02)	-6.60	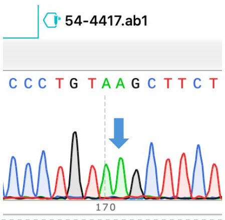	-7.93	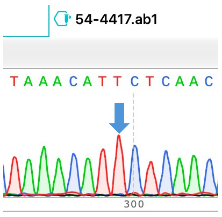	3.58	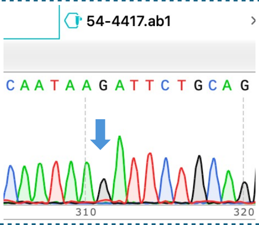
**Patient-59** G0/G2(20-20-02)	-4.63	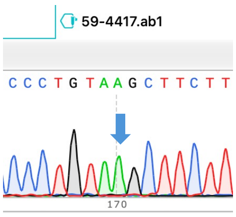	-3.54	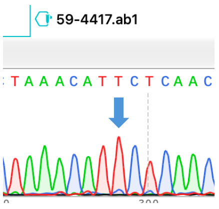	4.82	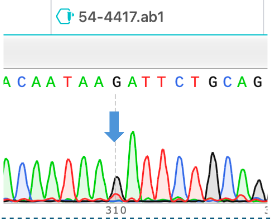

Supplementary Figure 4 Sanger histograms from 20 patients with diverse genetic background have been shown for validation of our qPCR result. Delta Ct values are shown on the left side for each SNP position together with their match histogram.

Supplementary Figure 5

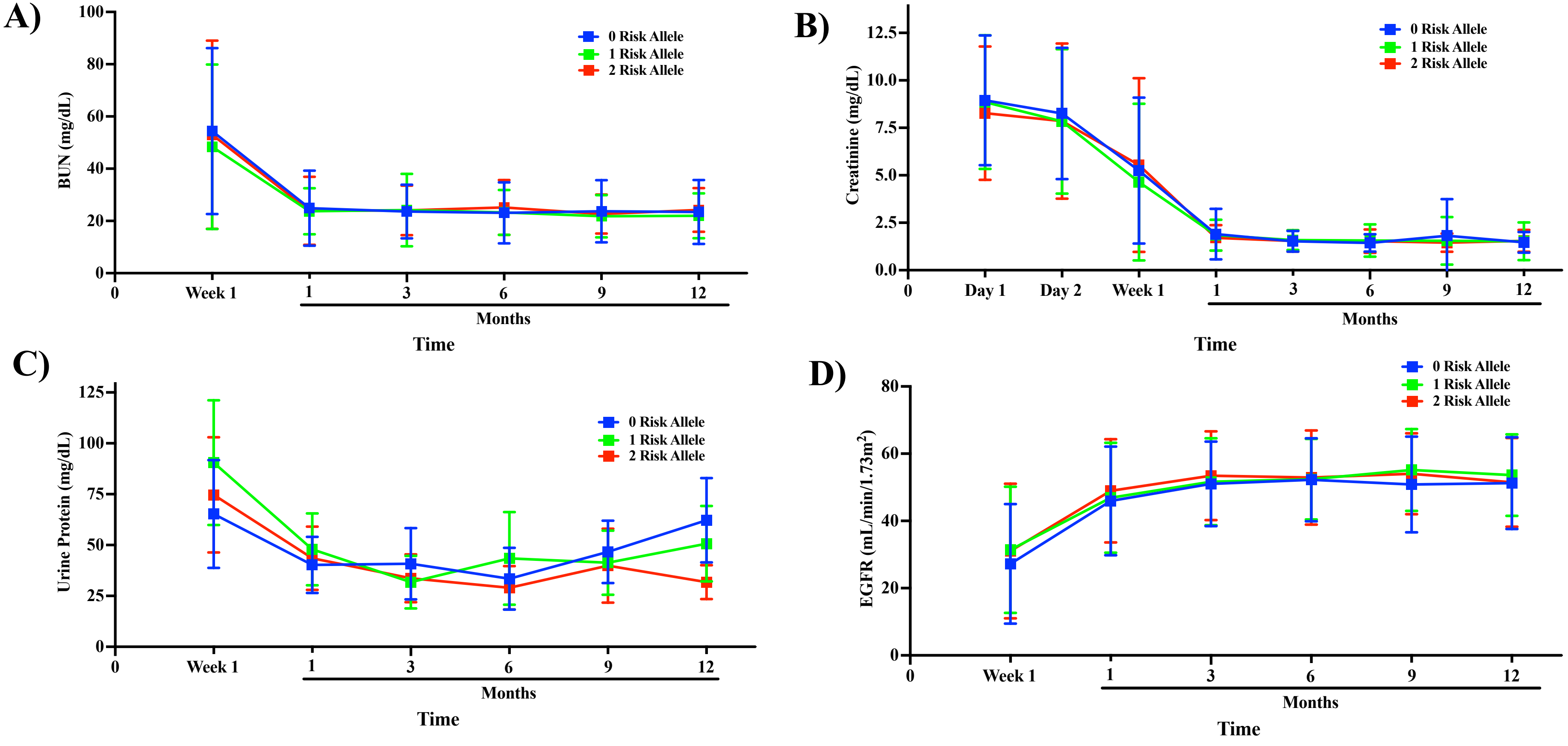

Supplementary Figure 5 Kidney function was studied in African American recipients based on risk allele carriage. There were no significant differences in kidney function among the groups.
